# MC4R Localizes at Excitatory Postsynaptic and Peri-Postsynaptic Sites of Hypothalamic Neurons in Primary Culture

**DOI:** 10.3390/cells13151235

**Published:** 2024-07-23

**Authors:** Haven Griffin, Jude Hanson, Kevin D. Phelan, Giulia Baldini

**Affiliations:** 1 Department of Biochemistry and Molecular Biology, University of Arkansas for Medical Sciences, Little Rock, AR 72205, USA; hgriffin@uams.edu (H.G.); jude.hanson733@gmail.com (J.H.); 2 Department of Neurobiology and Developmental Sciences, University of Arkansas for Medical Sciences, Little Rock, AR 72205, USA; phelankevind@uams.edu

**Keywords:** MC4R, postsynaptic sites, GPCR, hypothalamic neuron, primary culture, NMDAR, PSD95, neuron, primary culture, NMDA receptor

## Abstract

The melanocortin-4 receptor (MC4R) is a G protein-coupled receptor (GPCR) that is expressed in several brain locations encompassing the hypothalamus and the brainstem, where the receptor controls several body functions, including metabolism. In a well-defined pathway to decrease appetite, hypothalamic proopiomelanocortin (POMC) neurons localized in the arcuate nucleus (Arc) project to MC4R neurons in the paraventricular nuclei (PVN) to release the natural MC4R agonist α-melanocyte-stimulating hormone (α-MSH). Arc neurons also project excitatory glutamatergic fibers to the MC4R neurons in the PVN for a fast synaptic transmission to regulate a satiety pathway potentiated by α-MSH. By using super-resolution microscopy, we found that in hypothalamic neurons in a primary culture, postsynaptic density protein 95 (PSD95) colocalizes with GluN1, a subunit of the ionotropic N-methyl-D-aspartate receptor (NMDAR). Thus, hypothalamic neurons form excitatory postsynaptic specializations. To study the MC4R distribution at these sites, tagged HA-MC4R under the synapsin promoter was expressed in neurons by adeno-associated virus (AAV) gene transduction. HA-MC4R immunofluorescence peaked at the center and in proximity to the PSD95- and NMDAR-expressing sites. These data provide morphological evidence that MC4R localizes together with glutamate receptors at postsynaptic and peri-postsynaptic sites.

## 1. Introduction

The melanocortin-4 receptor (MC4R) is a G protein-coupled receptor (GPCR) that is expressed in several brain locations, including the hypothalamus and the brainstem, where the receptor controls several body functions, including metabolism [1,2,3,4]. In a well-defined pathway to control energy homeostasis, hypothalamic proopiomelanocortin (POMC) neurons localized to the arcuate nucleus (Arc) project to MC4R neurons in the paraventricular nuclei (PVN) to release the natural MC4R agonist α-melanocyte-stimulating hormone (α-MSH) to inhibit food intake [5,6,7,8,9]. At the ultrastructural level, the Arc POMC neurons extend axons that have boutons containing both neurotransmitter-containing small vesicles (SV) and neuropeptide-containing large vesicles (LV) to contact the dendrites of PVN neurons [5]. Arc neurons that express oxytocin receptors have been found to project excitatory glutamatergic fibers to the MC4R neurons in PVN for fast synaptic transmission to regulate the satiety pathway potentiated by α-MSH [10]. Glutamate neurotransmission is mediated by ionotropic glutamate receptors that include the N-methyl-D-aspartate receptor (NMDAR) and the α-amino-3-hydroxy-5-methyl-4-isoxazolepropionic acid receptors (AMPAR), which often function in synergy to mediate direct synaptic excitability and plasticity [11]. It has been found that the knockdown of MC4R in the PVN attenuates postsynaptic responses mediated by AMPAR by altering the phosphorylation of the GluA1 subunit of the receptor through the Gs/cyclic AMP (cAMP)/protein Kinase A (PKA) signaling pathway [12]. Together, the data suggest that synaptic connections between Arc neurons and PVN neurons to regulate satiety are mediated by glutamatergic signaling and are enhanced by MC4R. In another melanocortin system, MC4R expressed in the hippocampus is involved in cognition [1,2,3,4]. Within the hippocampal melanocortin system, POMC neurons in the CA3 region project to the MC4R neurons localized to the CA1 region. Also, in this system, MC4R signaling by Gs/cAMP/PKA signaling enhances postsynaptic glutamatergic neurotransmission by promoting the traffic of AMPARs to postsynaptic sites [3]. In the PVN of MC4R-GFP knock-in mice, MC4R tagged with GFP has been found to reside and function in cilia [13,14]. However, the ciliary localization of GPCRs directly controlling glutamatergic neurotransmission is unknown [15]. Differently, we have recently found that, in the hypothalamus of knock-in MC4R-HA mice, MC4R-HA with the HA tag placed at the C-terminus of the receptor is localized at the cell body and along neuronal processes of PVN neurons where the receptor reaches postsynaptic sites [16]. However, visualization of MC4R-HA required signal amplification, thus making it difficult to precisely assess the distribution of the receptor at these sites. Interestingly, super-resolution (SR) microscopy has been recently used as a tool to resolve the postsynaptic distribution of exogenous Metabotropic Glutamate Receptor Type 5 (mGluR5) in hippocampal neurons placed in primary culture at a nanoscale level [17]. Hypothalamic neurons derived from pre-adult mice and placed in primary culture have also been found to develop recurrent synapses and spines that express PSD95, the most abundant postsynaptic scaffold of excitatory synapses [18,19]. Here, we found that in murine hypothalamic neurons in primary culture, endogenous MC4R and exogenous HA-MC4R with the HA-tag placed at the N-terminus of the receptor distribute along the entire length of neuronal processes at discrete sites. By using SR microscopy, we found that HA-MC4R has dual distribution overlapping with and in the proximity of PSD95 and N-methyl-D-aspartate receptor (NMDAR) subunits GluN1 and GluN2A [20]. These data provide morphological evidence that MC4R resides together with the glutamate receptors at peri-synaptic and postsynaptic sites.

## 2. Materials and Methods

### 2.1. Chemicals

Formaldehyde (Cat # BP531-500), DMEM (Corning 10-013-CV, Hampton, NH, USA), penicillin/streptomycin 50X (Corning # 30-001-CI), L-glutamine (Corning 25-005-Cl, 2% *V*/*V*), and fetal bovine serum (FBS) (Atlas Biologicals # FP-0500-A, 10% *V*/*V*) were purchased. The Corning^®^ 100–1000 µL Universal Fit Racked Pipet Tips (Cat # 07-200-304), AccuTec Blades™ Personna Single-edge Prep Razor Blades (Cat # 12-640-18), Corning Falcon^®^ 70 µm cell strainer (Cat # 08-771-19), 18 × 18 mm Fisherbrand™ premium cover glasses (Cat # 12-548-AP), and Fisherbrand™ premium frosted microscope slides (Cat # 125442) were from Fisher Scientific. Neurobasal™-A Medium (Cat # 10888022), B-27™-Plus Neuronal Culture System (B27 Plus Neuro) (Cat # A3653401), RPMI 1640 Medium (Cat # 11875085) Lipofectamine™ 3000 Transfection Reagent (Cat # L3000015), Alexa Fluor™ 647 Tyramide SuperBoost™ Kit (goat anti-rabbit IgG, #B40926), and Hank’s Balanced Salt Solution (HBSS, Cat # 14170112) were purchased from ThermoFisher Scientific. Papain (Cat # LS0031191) and Earle’s Balanced Salt Solution (EBSS, Cat # LK003188) were purchased from Worthington Biochemical Corporation. The glass-bottomed 35 mm cell-culture dish (Cat # 801002) was from Southern Labware. Recombinant mouse FGF2 protein (Cat # ab229521) and recombinant human FGF1 protein (Cat # PHR1084 ab222361) were purchased from Abcam. Albumin from chicken egg white (Cat # A5503), and 1α,25-dihydroxyvitamin D3 (vitamin D3, Cat # D1530), DNase I (Cat # 11284932001), and Triton X-100 (Cat # X100-100ML) were from Millipore Sigma. Melanotan II (MTII, Cat # 2566) was from Tocris. NEB^®^ Stable Competent *E. coli* (High Efficiency) (Cat # C3040I) and NEBuilder^®^ HiFi DNA Assembly Master Mix (Cat E2621S) were from New England Biolabs. Primary antibodies: rabbit anti-PSD95 (Abcam #ab18258, 1:100 dilution), mouse anti-PSD95 (K28/43, NeuroMab 75-028, 1:200 dilution), mouse anti-PSD95 (Invitrogen MA1-046 1:25 dilution), rabbit anti-GluN1 (Alomone labs #AGC-001, 2 µg/mL final concentration), rabbit anti-NR2A/GluN2A (N327/95, NeuroMab 75-288, 1:50 dilution), rabbit anti-HA (C29F4, Cell Signaling Technology # 3724, 1:100 dilution), mouse anti-HA Tag (F-7) Alexa Fluor 647 (Santa Cruz Biotechnology, sc-7392 AF647, 1:100 dilution in live cells, 1:50 dilution in fixed cells). Rabbit anti-MC4R (MC4RIIIa, 12.4 µg/mL final concentration) and antigenic peptide were custom-made from Genscript. The secondary antibodies Alexa Fluor 488 goat anti-rabbit (Cat # 111-545-144), Cy™5 AffiniPure donkey anti-rabbit IgG (Cat # 711-175-152), Alexa Fluor 488 donkey anti-mouse (Cat # 715-545-151) were all used at a 1:100 dilution and were from Jackson Immuno Research Inc. The Neuro2A cells (Cat # CCL-131) were from ATCC. The Mc4r (NM_016977) Mouse Untagged Clone was from Origene (MC208946), and AAV-synapsin-Cre (AAV serotype 2) was from SignaGen Laboratories (Catalog SL101462).

### 2.2. Overexpression and Detection of Mouse MC4R and HA-MC4R in Neuro2A Cells

Neuro2A (Neuro 2A) cells were plated onto 3 cm plates and incubated for approximately 24 h in the complete DMEM media containing glutamine, penicillin/streptomycin, and FBS. On the next day, the cells were transiently transfected with 1 µg of either HA-MC4R or 2 µg of Mouse MC4R plasmids using Lipofectamine 3000, according to the manufacturer’s instructions, and incubated for 24 h. The transfected cells were then plated onto 3 cm polylysine-coated glass plates, incubated for 24 h, and fixed in 4% formaldehyde for 30 min, followed by three washes in PBS. The cells were permeabilized with permeabilizing immunofluorescence solution (PIF; 0.2% Triton, 100 µg/mL Ovalbumin, 0.01% Sodium Azide, and 1× PBS) for 30 min before primary antibody incubation. The Neuro 2A cells transfected with HA-MC4R were immunostained with an antibody against the HA tag (HA Rabbit), and the Neuro 2A cells transfected with untagged mouse MC4R were either immunostained with a custom antibody against the 20 amino acid peptide HIKRIAVLPGTGTIRQGTNC of mouse MC4R (MC4RIIIab) or were immunostained with MC4RIIIab that was pre-adsorbed in antigenic peptide (80 µg/mL final concentration) for 1 h. After 1 h of incubation in the primary antibodies, the cells were washed three times with PIF and incubated in secondary antibodies as follows: anti-rabbit 488 and anti-mouse 488. After 1h of incubation in the secondary antibodies, the cells were washed three times each with PIF and then with PBS and imaged on an Olympus Fluoview FV1000 microscope (Olympus Corporation, Tokyo, Japan).

### 2.3. Detection of Endogenous MC4R by Tyramide Amplification

The neurons were prepared the same as in “Preparation of primary hypothalamic neurons”. Endogenous MC4R was detected by incubating the primary neurons with and without MC4RIIIab. Tyramide amplification of MC4RIIIab was conducted according to the manufacturer’s instructions.

### 2.4. AAV2-Syn-HA-MC4R Plasmid Construction

The Synapsin-cyto-iATPSnFR1.0 plasmid was purchased from Addgene (Watertown, MA, USA Cat #102556) [21] and purified with the Promega Wizard^®^ Plus SV Minipreps DNA Purification System (Cat A1460). The plasmid was digested with BamHI and HindIII enzymes, and the 4.2 kb DNA band containing the synapsin promoter and AAV2 ITR regions was purified with the QIAquick Gel Extraction Kit (Cat # 28704). To insert hemagglutinin (HA)-tagged MC4R into the synapsin vector, we PCR-amplified the MC4R tagged with HA at the amino terminus (HA-MC4R) from the HA-MC4R-pEGFP-N2 plasmid, which is described previously [22]. PCR was conducted with forward and reverse primers (shown below) on the purified HA-MC4R fragment to generate a HA-MC4R insert with at least 20 bp overlapping regions homologous to the Synapsin vector. The PCR product was then run on a gel to confirm the amplification of the 1 kb DNA band containing HA-MC4R. The linearized Synapsin vector and HA-MC4R insert were then ligated with the NEBuilder HiFi DNA Assembly Master Mix (E2621) to obtain AAV2-Syn-HA-MC4R. Following assembly, competent *E. coli* cells were transformed with AAV2-Syn-HA-MC4R plasmid, and the DNA was purified as above. The correct insertion of HA-MC4R was verified by enzymatic digestion with HindIII and BamHI and ran on an ethidium bromide agarose gel to confirm the size of the HA-MC4R insert and Synapsin vector.

Forward primer: 5′- AAT TCA AGC TGC TAG CAA GGA TCC TAG CCA CCA TGT ATC CTT ATG ATG TG -3′
Reverse primer: 5′- CCC AGA GGT TGA TTA TCG ATA AGC TTC TTA ATA TCT GCT AGA CAA GTC ACA AAG GCC -3′

### 2.5. Preparation of Primary Hypothalamic Neurons

All solutions, sterilized by passage through 0.22-μm filters, were equilibrated with 95% O_2_ and 5% CO_2_. All glassware, plasticware, and instruments were sterile. Neurons from male and female hypothalami were pooled together. Brains derived from 4-week-old C57BL/6J mice were obtained from Jackson Laboratories (Cat # JAX:000664). Brains from CAMPER mice were also from Jackson Laboratories (Cat # JAX:032205) [23]. The brains were extracted and individually placed into a 24-well plate with 0.5 mL of ice-cold solution of Neurobasal A Medium containing 100U/mL of penicillin/streptomycin (Neurob-A P/S medium). For each brain, a section of an approximate 2 mm thickness was cut using a Stoelting stainless-steel brain matrix, with one single-edge blade placed anterior to the optic chiasm and the other blade placed anterior to the pons. From the coronal slice, a tissue rectangle of 2 mm width and 3 mm height centered around the entire third ventricle, including the mediobasal hypothalamus and the PVN, was dissected. The section was further cut into cubes of approximately 0.2 mm sides by using single-edge blades. The minced tissue was placed in an individual tissue-culture well containing 0.5 mL of EBSS with 20 U/mL of papain, and 1 mM EDTA and 1 mM DTT. The mixture was incubated in a tissue-culture cabinet equilibrated with 95% air and 5% CO_2_ for 15 min at 37 °C under agitation (OrbiShaker™ MP Orbital Microplate Shaker, 500 rpm), then DNAase was added from the 1% *w*/*v* stock solution in EBSS to reach a final concentration of 0.005% *w*/*v*, and the mixture was further incubated in the tissue-culture cabinet for other 15 min at 37 °C under continuous agitation. After digestion, FBS was added to reach a final concentration of 10% (*V*/*V*) to the mixture, which was passed once through a 1 mL pipet tip and pipetted into a cell strainer with 70 μm pores and then placed onto a 6-well plate. The filtered cells were pipetted onto glass-bottomed culture dishes pre-treated for 2 h with 0.5 mg/mL of poly-lysine (1–2 plates per hypothalamus). The plates were transferred to the cell-culture cabinet without agitation to allow for the cells to adhere to the glass bottom for 30 min. Then, 1 mL of RPM1-A was added to and removed twice from each plate to remove the unsedimented material. This was achieved by using a 1 mL pipette tip placed above the center of the coverslip while carefully leaving enough medium to cover the cells attached to the coverslip and, instead, removing all medium at the wall of the plate. The cells were incubated in 1 mL of B27 Plus Neuro medium containing 10 ng/mL of FGF1, 10 ng/mL of FGF2, 0.5 μg/mL of vitamin D3, 20 nM MTII, 100 IU penicillin, and 100 μg/mL of streptomycin (complete B27 Plus Neuro P/S). The cells were kept in the culture for 17–21 days by replacing 0.5 mL of the medium with 0.5 mL of fresh medium twice per week.

### 2.6. AAV2 Transduction

The conditions for the AAV2-dependent transduction of transgenes in primary hypothalamic neurons were initially established by using neurons from the CAMPER mouse line [23], where expression of brightly fluorescent cytosolic TEpacVV is dependent on the Cre recombinase. After about 9–10 days of in vitro (DIV) culture, the neurons were transduced with 3.5 µL of a 2 × 10^13^ vg/mL virus expressing Cre recombinase under the synapsin promoter (AAV2-synapsin-Cre). To transduce the cells, the media were quickly removed from the outside of the plate and replaced with 200 µL of neurobasal A containing 3.5 µL of a 2 × 10^13^ vg/mL virus. The neurons were incubated in the 200 µL of virus-containing media overnight, and the following morning, the medium was removed, and 1 mL of complete B27 Plus Neuro P/S was added to each plate. The neurons were fixed 8–10 days after transduction. AAV2-Syn-HA-MC4R particles expressing Syn-HA-MC4R were generated, purified, concentrated, and titered by the Viral Vector Core of the Emory Center for Neurodegenerative Disease Core Facilities.

### 2.7. Primary Neuron Immunostaining and Confocal Microscopy

The cells were permeabilized with PIF for 30 min. After permeabilization, the cells were incubated in primary antibodies for 1 h. After the primary antibody incubation, the cells were washed three times with PIF and incubated in secondary antibodies for 1 h. All primary and secondary antibodies were diluted in PIF. Following all antibody incubation, the cells were washed three times with PIF, twice with 1× PBS, and kept in 1× PBS at 4 °C until imaging. Immunostained neurons were then imaged on either the Olympus FV1000 microscope or the Zeiss LSM 880 confocal microscope (Carl Zeiss Microscopy, White Plains, NY, USA). Images taken on the Zeiss microscope using standard confocal microscopy were taken with a Plan-Apochromat 63×/1.4 Oil DIC M27 objective and a Z-stack interval of 1 µm.

### 2.8. Line Segment Analysis of Points of Endogenous MC4R and HA-MC4R Fluorescence

The confocal images taken with a 60× objective on the Olympus microscope were opened in ImageJ (2.14.0/1.54f; Java1.8.0_332) as OIF files and line segments were drawn along the individual points of endogenous MC4R fluorescence or HA-MC4R fluorescence using the straight-line tool. Using the Plot Profile function in ImageJ, fluorescence across the line was then measured as a function of gray values plotted over distance (in inches), and the values were exported to Excel (Version 2404 Build 16.0.17531.20190) for further analysis. The distance in inches was converted to pixels using the pixel width found in Image Properties in ImageJ. The distance in pixels was then converted to µm using the Olympus image dimensions. The maximum gray values across the lines of each central bright spot were then aligned. The gray values and their corresponding distances across the line were plotted in GraphPad Prism version 6.07 for Windows, GraphPad Software (La Jolla, CA, USA, www.graphpad.com) and displayed as the means +/− SD.

### 2.9. Airyscan Super-Resolution Microscopy

Airyscan imaging of the neurons was conducted using the Zeiss LSM 880 microscope with Airyscan detectors (Zen 2.3 SP1 FP3). The channel tracks were set to image fluorescence with 633 nm, 561, and/or 488 nm wavelength lasers and filters as follows: BP 570–620 + LP 645 for the 633 channel, BP 420–480 + BP 495–620 for the 561 channel, and BP 420–480 + BP 495–550 for the 488 channel. The optical thickness ranged from 0.3–0.5 µm, depending on the wavelength. For each sample, the Airyscan detectors were automatically aligned by selecting Airyscan in the Maintain tab of Zen Black 2.3/2.6 with the “Adjust in continuous scans” selected. The laser power, detector gains for each laser, and stage position were adjusted until the image acquisition status within the Airyscan tab was indicated as “Good (Waiting)”. Using an alpha Plan-Apochromat 100×/1.46 Oil DIC M27 Elyra objective with a 1.6 Optovar, 16-bit Airyscan images were taken in the SR mode with a Z-stack interval of 0.5 µm, a resolution of 1740 × 1740 pixels, and frame scan time of around 4 s. Following image acquisition, all images were processed by automatic 3D Airyscan processing. In Zen Blue 3.8 lite, a single frame in which the neurites appeared most in focus was selected from the Z-stack, and the Airyscan-processed image was sharpened using the Enhance Contour method in the Processing tab with the parameters set to process in the Z-dimension with a strength of 1 and automatic normalization.

### 2.10. Measuring the Diameter of PSD95 Points

The Airyscan images were opened in ImageJ (2.14.0/1.54f; Java1.8.0_332) as TIF files and line segments were drawn along the individual points of PSD95 fluorescence using the straight-line tool. For each cell, a single line was used to measure the fluorescence across individual PSD95 points, with 30 points per cell. Using the Plot Profile function in ImageJ, fluorescence across the line was then measured as a function of gray values plotted over distance (in inches), and the values were then exported to Excel for further analysis. The distance in inches was converted to pixels using the pixel width found in Image Properties in ImageJ. The distance in pixels was then converted to µm using the Zeiss image dimensions (1740 × 1740 pixels, 46.59 µm × 46.59 µm), averaging about 37 pixels/µm. The maximum gray values across the 30 lines of each central bright spot were then aligned. The average 25% of the maximum gray values were then determined and used to define the fluorescence values within the central bright spot of PSD95. The distance (in µm) along the fluorescence values greater than 25% of the maximum gray values was measured to determine the diameters and radii of the PSD95 points for each cell. The diameters of the PSD95 points for each cell analyzed were then averaged and divided by 2 to determine the average radius of the PSD95 points of fluorescence in our samples. The average radius size of PSD95 was then later used to determine the maximum distance between two points in which they would still be considered as colocalizing.

### 2.11. Line Segment Analyses

Airyscan images were opened in ImageJ as TIF files, and line segments were drawn using the straight-line tool. Three lines were drawn in different orientations, with the center of each line crossing a bright spot of fluorescence. Using the Plot Profile function in ImageJ, the fluorescence signals in channels 1 and 2 across the line were then measured as a function of gray values plotted over distance (in inches), and the values were then exported to Excel for further analysis. The distance in inches was converted to pixels using the pixel width found in Image Properties in ImageJ. The distance in pixels was then converted to µm using the Zeiss image dimensions. The maximum gray values across the three intersecting lines of the central bright spot were then aligned, and the gray values of fluorescence for each line in the other channel were then re-positioned along the line distance to match the corresponding values in the other channel. The gray values across their corresponding distances across each of the three lines were plotted in GraphPad Prism version 6.07 for Windows and displayed as the means +/− SEM. The peaks of fluorescence were defined as the maximum gray value of at least 2× greater than the baseline gray values. The distance between the fluorescence peaks in channel 1 and channel 2 was separated into two groups: those that were less than 216 nm (colocalizing) and those that were greater than the average radius of the PSD95 points (adjacent).

### 2.12. Statistical Analysis

All statistical analyses were performed using GraphPad Prism 6 software. The results for the hypothalamic neurons were derived using at least 2–6 Petri plates in 2–3 independent experiments for each postsynaptic marker. The column graphs show the data as means +/− SD; column analysis was carried out by using a 95 percent confidence interval. The XY graphs show the data as means +/− SEM.

## 3. Results

### 3.1. Hypothalamic Neurons in Primary Culture Express GluN1 Localized at Postsynaptic Sites

**Figure 1:**
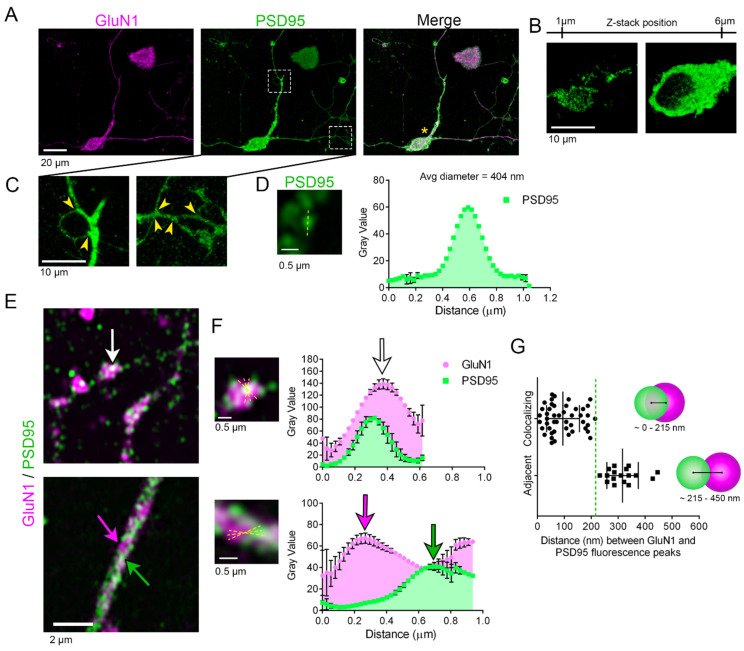
Hypothalamic neurons in primary culture express GluN1 localized at postsynaptic sites. (**A**) An orthogonal projection of six confocal images of a primary neuron expressing endogenous GluN1 and PSD95 visualized with the rabbit anti-GluN1 (AGC-001) and anti-PSD95 K28/43 antibody, respectively. The white rectangles indicate the inset regions shown at higher magnification in (**C**), and the yellow asterisk indicates the neuron of interest. (**B**) Left and right panels: Single confocal images from different positions of a Z-stack of 6 µm thickness showing the punctate distribution of PSD95 (left) and the negative staining of the nucleus (right) in the cell soma of the neuron shown in (**A**). (**C**) Enlarged images of the inset regions in (**A**). Yellow arrowheads indicate branching points. (**D**) A representative SR image of a point of PSD95 fluorescence with a representative yellow dashed line was used to determine the average diameter of PSD95 points. The graph on the right illustrates the average +/− SEM fluorescence (gray value) of PSD95 along the line, determined by measuring 180 spots of PSD95 fluorescence from 6 neurons. (**E**,**F**) SR images of a neurite expressing GluN1 and PSD95. The white arrow indicates a spot of PSD95 and GluN1 colocalization, and the green/magenta arrows indicate spots where PSD95 and GluN1 are adjacent. The regions indicated by the arrows are shown enlarged in (**F**). Yellow lines indicate those drawn for line segment analyses, with corresponding graphs displayed as means +/− SEM. (**G**) The distance (nm) between GluN1 and PSD95 fluorescence peaks was determined by the line segment analyses from 6 neurons from 2 independent experiments (n = 46 colocalizing points, n = 17 adjacent points), displayed as means +/− SD. The green dotted line is placed at the 216 nm cutoff that discriminates spots at which GluN1 and PSD95 colocalize (distance of fluorescence peaks < 216 nm) and those where GluN1 and PSD95 are adjacent (distance between fluorescence peaks > 216 nm and <450 nm), as indicated by the models.

At the excitatory synapses, the PSDs are dense, localized areas at the top of the spines, where the most abundant protein is PSD95 [24]. When hypothalamic neurons derived from pre-adult mice are placed in primary cultures for 2–3 weeks, they extend microtubule-associated protein 2 (MAP2 positive) dendrites with spines that express PSD95 [18]. Single hippocampal neurons can form synapses, including excitatory synapses, exclusively with themselves (autapses), thereby providing an experimental system that allows both morphological and functional experiments [25,26,27]. Post-natal hypothalamic neurons also form functional autapses as they respond to evoked action potentials by the generation of autaptic currents that can be inhibited by the GABA(A) receptor antagonist [18]. MTII was added to the primary culture of hypothalamic neurons in addition to FGF1 and FGF2 because we found that the MC4R agonist promotes the formation of long processes in neurons that can differentiate into cholinergic and Sim1/MC4R neurons [19]. Glutamatergic neurons in primary cultures have been used to study the role of PSD95 in the stabilization and desensitization of NMDAR [28,29]. Confocal microscopy of the primary hypothalamic culture finds that virtually every cell in the field expresses the NMDAR subunit GluN1 and PSD95 (orthogonal projection of Figure 1A). However, some cells did not form any processes and, nevertheless, expressed the neuronal markers GluN1 and PSD95. Other cells (yellow asterisk, Figure 1A) expressed GluN1 and PSD95, which appeared as puncta at the cell body (Figure 1A,B) and extended branched processes (Figure 1C). The Zeiss Airyscan microscope transforms a diffraction-limited confocal microscope with a resolution of ~250 nm into a super-resolution microscope with a resolution of ~120 nm by using a 32-channel detector [30]. Using super-resolution microscopy, PSD95 appeared at the neuronal processes of hypothalamic neurons as puncta with an average diameter of 404 nm (Figure 1D, n = 180 puncta analyzed, n = 6 neurons, SD = 133 nm, and lower and upper 95% CI of mean = 384 and 423 nm, respectively), which is consistent with the previously described <1 μm size of PSDs [31]. PSD95 functions in the recruitment and stabilization of iGluRs [24,32]. By electron microscopic immunogold analysis and super-resolution microscopy, NMDAR localizes to the center of postsynaptic sites together with PSD95 [33,34], which is consistent with immunoprecipitation studies finding that GluN1 interacts with PSD95 [35,36,37]. We reasoned that if NMDAR localized to the postsynaptic sites in primary hypothalamic neurons, then peaks of GluN1 immunofluorescence would exist at a close distance, approximately within the average radius (the distance was set at 216 nm) of PSD95 immunoreactivity. The segment analysis shows that peaks of GluN1 immunofluorescence could be found in proximity with those of PSD95 (n = 46 spots analyzed, n = 6 neurons, mean distance of GluN1 and PSD95 peaks = 94.96 nm, SD = 60.9 nm, and lower and upper 95% CI of mean = 78.88 and 113 nm, respectively) (Figure 1E–G). Thus, hypothalamic neurons form excitatory postsynaptic specializations that include PSD95 and GluN1. Peaks of GluN1 and PSD95 immunostaining also appeared at a higher distance than 216 nm (n = 17 spots analyzed, the mean distance of GluN1 and PSD95 peaks = 316.2 nm, SD = 58.1 nm, and lower and upper 95% CI of mean = 286.3 and 346 nm, respectively), indicating the peri-postsynaptic localization of NMDAR. The peri-postsynaptic localization of GluN1 is consistent with postsynaptic NMDAR being endocytosed and trafficked to degradative compartments [38].

### 3.2. Murine Endogenous MC4R and Human Epitope-Tagged HA-MC4R Have a Similar Distribution in Primary Hypothalamic Neurons

**Figure 2:**
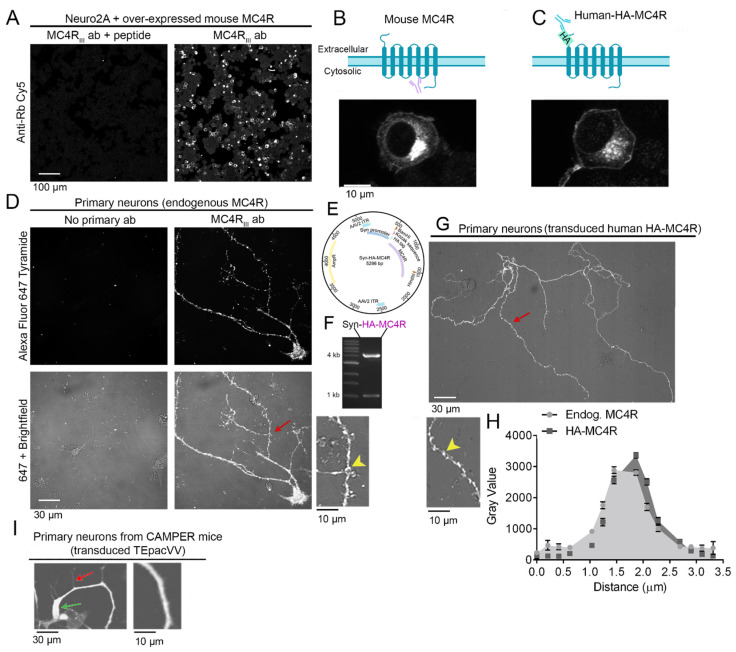
Murine endogenous MC4R and human epitope-tagged HA-MC4R have a similar distribution in primary hypothalamic neurons. (**A**) Confocal images of Neuro2A cells that were transiently transfected with mouse MC4R and were incubated with MC4RIIIab that was either pre-adsorbed with antigenic peptide (left) or not pre-adsorbed (right). Scale bar: 100 µm. (**B**) Top panel: Illustration of untagged mouse MC4R and the MC4RIIIab antibody binding to the third intracellular loop of the receptor. Bottom panel: Confocal image of a Neuro2A cell transiently transfected with mouse MC4R and immune-stained with MC4RIIIab. (**C**) Top panel: Illustration of human MC4R containing the HA tag at the N-terminus and the HA antibody binding to the tag. Bottom panel: Confocal image of a Neuro2A cell transiently transfected with HA-MC4R and immune-stained with the HA antibody. (**D**) Confocal images of primary neurons incubated with (right) or without (left) MC4RIIIab and visualized by tyramide amplification. Top panel: 647 nm fluorescence only; bottom panel: 647 nm fluorescence merged with the brightfield. The region indicated by the red arrow is enlarged to indicate individual spots of endogenous murine MC4R fluorescence (yellow arrowhead). (**E**) Diagram of the AAV2-Syn-HA-MC4R plasmid. (**F**) Gel electrophoresis of the AAV2-Syn-HA-MC4R plasmid, digested with HindIII and BamHI, showing the vector backbone migrating at the 4 kb band of DNA ladder, and the HA-MC4R insert, migrating at the 1 kb band of DNA ladder. (**G**) Live cell confocal imaging of a neuron transduced with AAV2-Syn-HA-MC4R and incubated with mouse anti-HA Tag (F-7) conjugated to Alexa Fluor 647 for 30 min before imaging. The region indicated by the red arrow is enlarged to indicate individual spots of exogenous HA-MC4R fluorescence (yellow arrowhead). (**H**) Line segment analyses of n = 49 spots of endogenous MC4R immunofluorescence and n = 42 spots for HA-MC4R immunofluorescence, displayed as means +/− SEM. (**I**) CAMPER neurons transduced with AAV2-synapsin-Cre expressing cytosolic TEpacVV, appearing as diffuse fluorescence in the cell body and (green arrow) in the primary process is shown enlarged in the right panel. A secondary process is indicated by a red arrow.

In the murine paraventricular region (PVN) of the hypothalamus, Sim1/MC4R neurons express MC4R [16,39,40]. Our previous work finds that in coronal slices of knock-in mice expressing MC4R-HA, the receptor could be clearly detected in the PVN by using immunostaining with anti-HA antibodies in combination with tyramide signal amplification (TSA) [16]. However, detection of endogenous MC4R is difficult, likely because the protein is covalently modified by glycosylation at multiple asparagine residues at the N-terminus and serine–threonine sites at the carboxyl terminus [41,42]—and most available antibodies target these domains. To visualize the untagged MC4R, we newly generated an antibody against the third intracellular loop of murine MC4R (MC4R_III_ab). Neuro2A cells were transfected with the murine untagged MC4R, and immunostaining was carried out by using MC4R_III_ab. Murine exogenous MC4R appeared at the perinuclear region and at the margin of Neuro2A cells, like human exogenous HA-MC4R bearing a HA-tag at the amino terminus (Figure 2A–C). Conversely, cells treated in parallel with MC4R_III_ab that were pre-incubated with the same peptide used to raise the antibody did not exhibit any signal (Figure 2A). Thus, MC4R_III_ab can specifically detect overexpressed untagged murine MC4R. However, when MC4R_III_ab was used for the detection of endogenous MC4R in primary hypothalamic neurons, the signal was almost undetectable. This is consistent with MC4R being expressed in low abundance, similar to that of mGluR expressed in neurons [17,43]. Differently, when the MC4R_III_ab immunostaining of primary neurons was carried out in combination with TSA, MC4R was clearly detectable in the cell body and along the entire length of primary, secondary, and tertiary neuronal processes (Figure 2D). At the processes, MC4R appeared as discrete puncta, suggesting postsynaptic distribution (yellow arrowhead). In the TSA reaction, side-chains of proteins adjacent to the MC4R_III_ab/MC4R complex are covalently linked to the fluorescent tyramide, thereby providing high-density immunolabeling in the proximity of, rather than coincident with, MC4R. We then asked whether exogenous human HA-MC4R over-expressed in primary neurons replicated the punctate distribution of the endogenous receptor along the entire arborization of the processes. To express human HA-MC4R under the synapsin promoter, HA-MC4R cDNA was subcloned into an Adeno-Associated Virus Type2 (AAV2) vector under the synapsin promoter (Figure 2E,F) and packaged into AAV2-Syn-HA-MC4R viral particles. The synapsin promoter was chosen to express HA-MC4R because of the high specificity of transgene expression to neurons [44,45]. When the hypothalamic neurons were transduced with AAV2-Syn-HA-MC4R particles and the live cells were immune-stained by using the anti-HA antibody in the absence of signal amplification, the receptor appeared as discrete puncta again (red arrow and yellow arrowhead, Figure 2G) along the entire arborization of the neuronal processes. The line segment analysis found that the puncta of endogenous MC4R (n = 49 spots analyzed) and HA-MC4R (n = 42 spots analyzed), imaged by confocal fluorescence, have similar diameters (Figure 2H). Conversely, in CAMPER neurons transduced with AAV2-synapsin-Cre, the cytosolic TEpacVV appeared as a diffuse fluorescence in the cell body and processes (Figure 2I). Thus, HA-MC4R replicates the punctate distribution of endogenous MC4R at distinct spots along the entire length of the neuronal processes of primary hypothalamic neurons.

### 3.3. HA-MC4R Delivered to Hypothalamic Neurons by AAV2 Distributes across the Entire Cytoplasm of the Cell Body and along Neurites, Where It Colocalizes with PSD95

**Figure 3:**
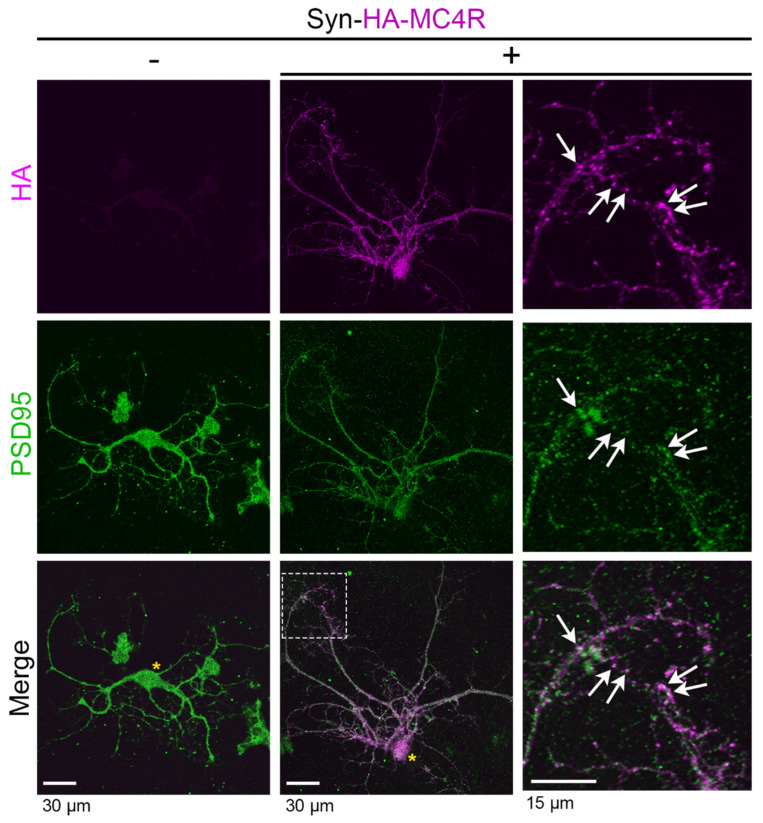
HA-MC4R expressed at neuronal processes colocalizes with PSD95. Confocal images of neurons treated without AAV2 (left panel) and neurons transduced with AAV2 Syn-HA-MC4R (middle and right panels). Neurons were immunostained with rabbit anti-HA C29F4 antibodies against the HA tag and mouse anti-PSD95 MA1-046 against PSD95. The yellow asterisks indicate the cell body, the white rectangle indicates the region shown enlarged in the right panels, and the white arrows indicate points of colocalization.

In the coronal slices of knock-in MC4R-HA mice, murine MC4R-HA bearing the HA-tag at the C-terminus of the receptor colocalized with PSD95 along the neuronal processes suggests postsynaptic distribution [46]. When the primary hypothalamic neurons transduced with AAV2-Syn-HA-MC4R particles were visualized by confocal microscopy, HA-MC4R was also colocalized with PSD95 (Figure 3 white arrows). Conversely, the neurons that were not transduced with AAV2 did not have any specific HA immunostaining. Thus, the HA-MC4R overexpressed in primary hypothalamic neurons in primary culture replicates the postsynaptic distribution of MC4R-HA in the PVN neurons [46].

### 3.4. MC4R-HA Localizes at Postsynaptic and Peri-Postsynaptic Sites

**Figure 4:**
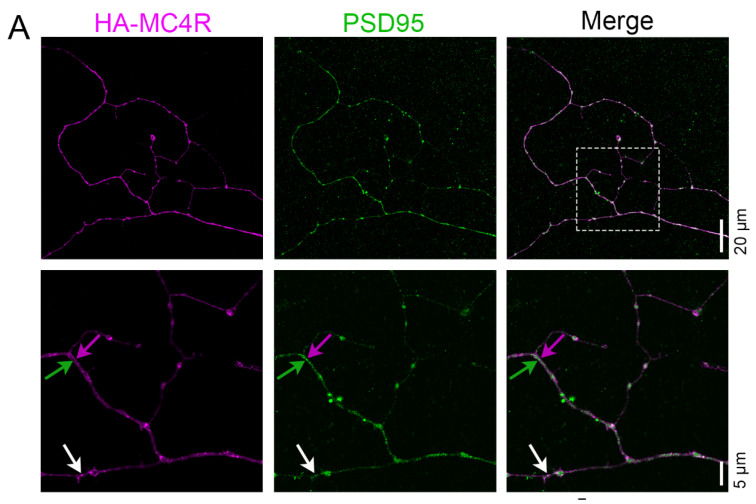

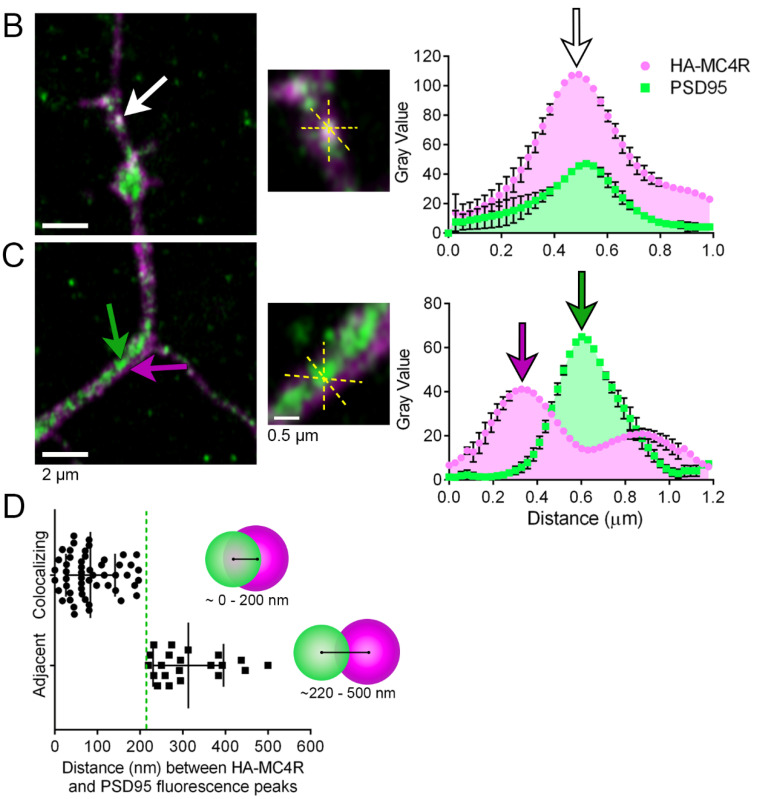
HA-MC4R localizes at the postsynaptic and peri-postsynaptic sites. (**A**) Confocal images (20 µm scale bar) and SR images (5 µm scale bar) of a HA-MC4R-expressing neuron, immunostained with rabbit anti-HA C29F4 antibodies and mouse anti-PSD95 MA1-046. The white rectangle indicates the enlarged region. The white arrow indicates a point of colocalization, and the green/magenta arrows indicate a spot where PSD95 and HA-MC4R are adjacent. (**B**) Enlarged images of the neurite are shown above, where HA-MC4R and PSD95 colocalize (white arrow). (**C**) Enlarged images of the neurite above where HA-MC4R and PSD95 are adjacent (magenta and green arrows). (**B**,**C**) The yellow dashed lines indicate those drawn for the segment analyses, with the corresponding graphs displayed as means +/− SEM on the right. (**D**) The distance (nm) between HA-MC4R and PSD95 fluorescence peaks was determined by line segment analyses from 5 neurons from 3 independent experiments (n = 52 colocalizing points, n = 21 adjacent points) displayed as the means +/− SD. The green dotted line in the graph is as in Figure 1. Models indicate the range of distances between colocalizing and adjacent peaks of HA-MC4R and PSD95 fluorescence, respectively.

GPCRs that participate in glutamatergic signals rather than localize at postsynaptic sites are found in the vicinity of such sites [31]. With this respect, metabotropic glutamate receptors (mGluRs) are largely excluded from the PSDs and instead concentrate at annular peri-synaptic rings of 100–200 nm thickness around a center where NMDAR and AMPAR are often co-expressed [31,47,48,49,50,51]. To determine whether MC4R localizes at the postsynaptic and/or peri-postsynaptic sites, HA-MC4R immunofluorescence in the neuronal processes was imaged by Airyscan super-resolution microscopy (Figure 4A). By using the segment analysis ImageJ software tool, the HA-MC4R and PSD95 fluorescence intensities peaked within 216 nm (Figure 4B, white arrow, and Figure 4D, n = 52 spots analyzed, n = 5 neurons, mean distance of HA-MC4R and PSD95 peaks = 83.47 nm, SD = 57.52 nm, and lower and upper 95% CI of mean = 67.46 and 99.48 nm, respectively), indicating MC4R residency at the center of the postsynaptic sites. Instead, at other spots, peaks of HA-MC4R and PSD95 were more separated (>216 nm distance) but still existed within 500 nm (Figure 4C, green and magenta arrows, and Figure 4D, n = 21 spots analyzed, the mean distance of HA-MC4R and PSD95 peaks = 312.8 nm, SD = 82.5 nm, and lower and upper 95% CI of mean = 275.3 and 350.4 nm, respectively). Thus, these data indicate that MC4R is expressed both at the postsynaptic and peri-postsynaptic sites.

### 3.5. MC4R-HA Localizes Together with and in the Proximity of GluN1 and GluN2A

**Figure 5:**
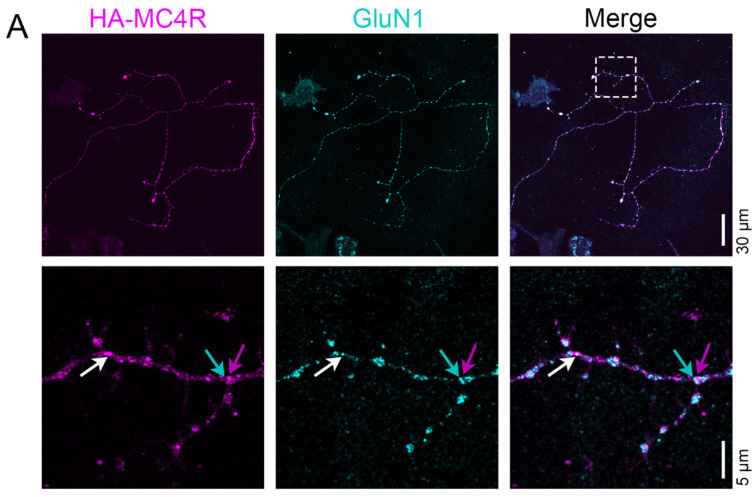

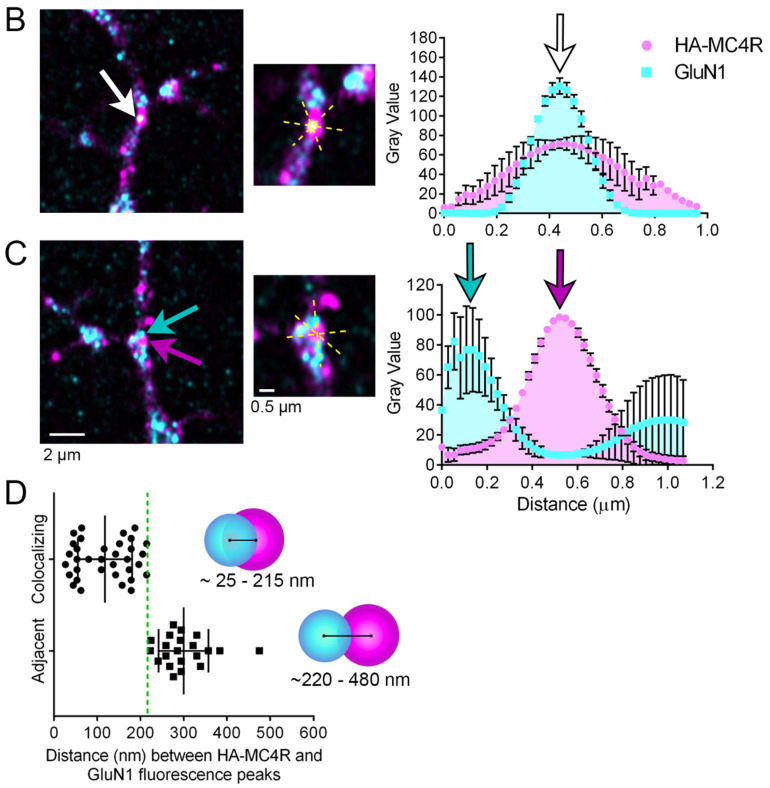
HA-MC4R localizes together with and in the proximity of GluN1. (**A**) Confocal images (30 µm scale bar) and SR images (5 µm scale bar) of an HA-MC4R-expressing neuron, immunostained with antibodies with mouse anti-HA Tag (F-7) and rabbit anti-GluN1 (AGC-001) to visualize endogenous GluN1. The white rectangle indicates the enlarged region. The white arrow indicates a point of colocalization, and the cyan/magenta arrows indicate a site where GluN1 and HA-MC4R are adjacent. (**B**) Enlarged images of the neurite above where HA-MC4R and GluN1 colocalize (white arrow). (**C**) Enlarged images of the neurite above where HA-MC4R and GluN1 are adjacent (magenta and cyan arrows). (**B**,**C**) The yellow dashed lines indicate those drawn for the segment analyses, with the corresponding graphs displayed as means +/− SEM on the right. (**D**) The distance (nm) between HA-MC4R and GluN1 fluorescence peaks was determined by line segment analyses from 3 neurons from 2 independent experiments (n = 34 colocalizing points, 21 adjacent points). The green dotted line in the graph is as in Figure 1. Models indicate the range of distances between colocalizing and adjacent peaks of HA-MC4R and GluN1 fluorescence, respectively.

**Figure 6:**
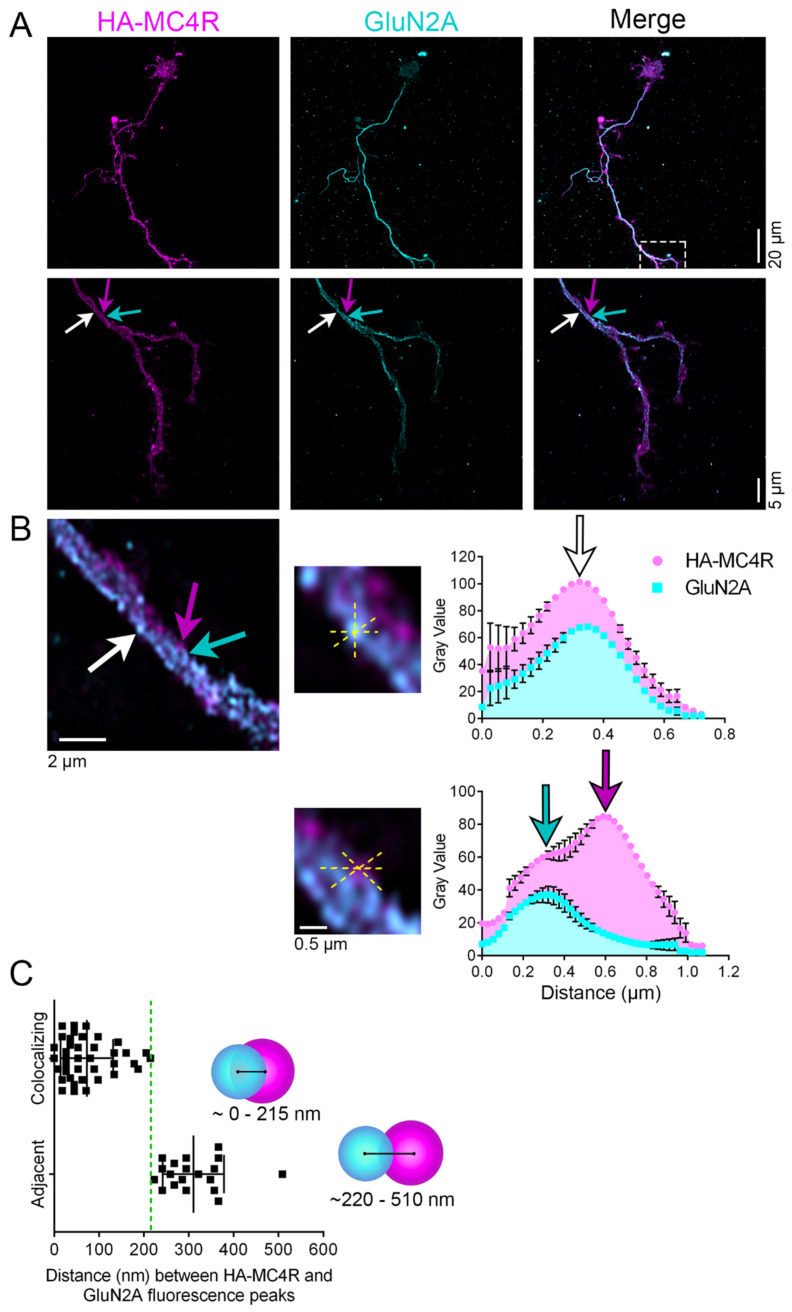
HA-MC4R localizes together with and in the proximity of GluN2A. (**A**) Confocal images (20 µm scale bar) and SR images (5 µm scale bar) of an HA-MC4R-expressing neuron, immunostained with mouse anti-HA Tag (F-7) and anti-NR2A (N327/95) to visualize endogenous GluN2A. The white rectangle indicates the relative region of the enlarged images in the panel below. The white arrow indicates a point of colocalization, and the cyan/magenta arrows indicate a site where GluN2A and HA-MC4R are adjacent. (**B**) Enlarged images of the neurite above where HA-MC4R and GluN2A colocalize (white arrow) and where they are adjacent (cyan/magenta arrow). The yellow dashed lines indicate those drawn for the segment analyses, with the corresponding graphs displayed as means +/− SEM on the right. (**C**) The distance (nm) between HA-MC4R and GluN2A fluorescence peaks was determined by line segment analyses from 5 neurons from 2 independent experiments (n = 39 colocalizing points, n = 19 adjacent points). The green dotted line in the graph is as in Figure 1. Models indicate the range of distances between colocalizing and adjacent peaks of HA-MC4R and GluN2A fluorescence, respectively.

NMDAR localizes to the center of the postsynaptic sites together with PSD95 [33,34]. In the melanocortin pathway, a population of Arc neurons project excitatory glutamatergic fibers to the MC4R neurons in the PVN for fast synaptic transmission to regulate a satiety pathway potentiated by α-MSH [10]. Thus, we asked whether MC4R and NMDAR can reside together at the postsynaptic sites. NMDAR are hetero-tetramers that include two obligatory glycine-binding GluN1 subunits together with two other glutamate-binding subunits, such as GluN2A [20]. When neurons transduced with AAV2-Syn-HA-MC4R were co-stained with anti-HA and anti-GluN1 (Figure 5A), peaks of HA-MC4R and GluN1 immunofluorescence appeared within 216 nm (Figure 5B, white arrow, and Figure 5D, n = 34 spots analyzed, n = 3 neurons, the mean distance of HA-MC4R and GluN1 peaks = 118.2 nm, SD = 62.47 nm, and lower and upper 95% CI of mean = 96.42 and 140 nm, respectively). At other spots, peaks of HA-MC4R and GluN1 immunofluorescence were separated by more than 216 nm but still existed within 480 nm (Figure 5C, green and magenta arrows, and Figure 5D, n = 21 spots analyzed, the mean distance of HA-MC4R and GluN1 peaks = 299.7 nm, SD = 57.72 nm, and lower and upper 95% CI of mean = 273.4 and 326 nm, respectively). When neurons transduced with AAV2-Syn-HA-MC4R were co-stained with anti-HA and anti-GluN2A (Figure 6A), peaks of HA-MC4R and GluN2A immunofluorescence again appeared both within 216 nm (Figure 6B,C, white arrow, n = 39 spots analyzed, n = 5 neurons, the mean distance of HA-MC4R and GluN2A peaks = 73 nm, SD = 58.81 nm, and lower and upper 95% CI of mean = 53.94 and 92.07 nm, respectively) and were separated by more than 216 nm but were still within 510 nm (Figure 6B,C, green and magenta arrows, n = 19 spots analyzed, the mean distance of HA-MC4R and GluN2 peaks = 310.5 nm, SD = 68.4 nm, and lower and upper 95% CI of mean = 277.5 and 343.5 nm, respectively). These data indicate that MC4R and NMDAR subunits can exist at the same spot, suggesting that functional cooperation between the two receptors may be promoted by MC4R delivery at the postsynaptic sites.

## 4. Discussion

Nanoscale analysis of MC4R localization finds the receptor at postsynaptic and peri-postsynaptic sites that express NMDAR and PSD95 along processes of hypothalamic neurons. Though many studies have shown the localization of type-C GPCRs, such as mGluRs, to glutamatergic postsynaptic sites [31,52], only a few have demonstrated the localization of type-A GPCRs at such sites [53,54], and none of them are by SR microscopy.

Localization of MC4R at the puncta along neuron processes relies on the following evidence: (1) immunofluorescence confocal microscopy using the new MC4R_III_ab antibody finds that endogenous murine MC4R localizes to the puncta along the entire arborization of the neuronal processes of primary hypothalamic neurons; (2) immunofluorescence confocal microscopy, by using two different commercially available antibodies against the HA-tag of overexpressed HA-MC4R, also localizes to the puncta with the same size along the entire length of the neuronal processes; and (3) differently, a cytosolic protein, TEpacVV, expressed by Cre recombinase under the synapsin promoter, has a diffuse distribution. The specificity of immunostaining by the MC4R_III_ab to detect mouse MC4R is controlled by the antibody being able to detect exogenous MC4R in the Neuro2A cells only when the antibody is not pre-adsorbed to the antigen. The specificity of immunostaining by the anti-HA antibodies is controlled by the signal being dependent on the transduction of HA-MC4R in primary neurons. By using SR microscopy, we find that the puncta of HA-MC4R immunostaining colocalize with and are in the vicinity of PSD95 and NMDAR subunits GluN1 and GluN2, indicating residency of the GPCR at postsynaptic and peri-synaptic sites. The reproducibility of distribution of MC4R in the proximity of postsynaptic markers is confirmed by finding that peaks of MC4R are found within the average distances of 70–120 nm from those of PSD95, GluN1, and GluN2 across a total of 125 spots derived from 19 different neurons. The postsynaptic distribution of MC4R is consistent with earlier results, where MC4R-HA with the HA tag at the C-terminus of the receptor, expressed under the endogenous promoter, was found to localize along the neuronal processes of PVN neurons at postsynaptic sites of brain slices [16]. Thus, HA-MC4R expressed in primary hypothalamic neurons has the same punctate distribution along the neuronal processes as the endogenous MC4R.

PVN MC4R neurons are necessary and sufficient to regulate feeding by extending fibers that reach the parabrachial nucleus [39,55]. Food presentation can, by itself, rapidly modulate the activity of POMC and AgRP neurons to control appetitive behavior [56]. Arc neurons that express oxytocin receptors have been found to project excitatory glutamatergic fibers to the PVN for fast synaptic transmission to regulate satiety by a pathway potentiated by α-MSH [10]. Thus, synaptic connections between the Arc neurons and PVN neurons, mediated at least in part by glutamate and enhanced by MC4R signaling, may function as a rapid circuit to regulate satiety. Functional studies have found that the knockdown of MC4R in the PVN can attenuate the postsynaptic response of AMPAR by promoting phosphorylation of the GluA1 subunit through the GαS/PKA-dependent signaling cascade and lead to rapid body weight gain [12]. Similarly, an earlier report found that, within the hippocampal melanocortin system, MC4R regulates hippocampal synaptic plasticity by a PKA-based signal to promote the surface expression of GluA1-containing AMPARs [57]. These studies converge to indicate the role of MC4R in modulating glutamatergic neurotransmission. Consistent with that, our earlier studies found that in the PVN of knock-in mice, tagged MC4R-HA expressed under the physiological promoter colocalizes with PSD95, a marker of excitatory postsynaptic sites [16]. However, precise postsynaptic localization of MC4R distribution in the brain remains difficult because MC4R immunoreactivity in the MC4R-HA knock-in mice could be visualized only by using an enzymatic step to amplify the signal [16]. To visualize MC4R localization in primary hypothalamic neurons, we have used two approaches as follows: (1) Detection of the endogenous receptor by using a novel antibody raised against murine MC4R. This approach again required signal amplification, thereby making nanoscale localization of the receptor by SR microscopy difficult to carry out. (2) AAV2-mediated expression of human-tagged HA-MC4R under the synapsin promoter. The synapsin promoter allows a strong expression of transgenes in neurons by an adenovirus [44,45], a condition that facilitates protein localization by SR microscopy [58]. However, protein overexpression may induce protein mis-localization. Importantly, we found that both endogenous MC4R and over-expressed HA-MC4R are localized at discrete spots with similar sizes along the entire length of the neuronal processes. Thus, synapsin promoter-driven over-expression of tagged MC4R does not appear to change the distribution of the receptor along the neuronal processes. Furthermore, AAV2-dependent delivery of HA-MC4R allows for the precise identification of receptor membrane distribution at the nanoscale level because the non-amplified immunostaining signal coincides with MC4R rather than existing on side-chains of proteins in the proximity of the receptor. The finding by SR microscopy that MC4R can localize at the same postsynaptic sites that express GluN1, GluN2A and PSD-95 is consistent with data from other investigators that MC4R agonism functions to potentiate synaptic excitatory neurotransmission [10,12,57].

mGluR1 and mGluR5 are type-C GPCRs that accumulate at an annular ring of ~200 nm thickness that constitutes the peri-postsynaptic zone to modulate calcium responses [47,50,51,59]. Integral to PSD glutamatergic signaling is the reciprocal interplay between GluN1 and mGluR5 [17,60,61]. By using SR microscopy, we newly found that MC4R, which is a type-A GPCR, has a dual synaptic distribution by being able to accumulate both at the center of the PSD, together with NMDAR subunit GluN1, and at the peri-postsynaptic site. POMC and AgRP neurons have axons that have synaptic boutons where neurotransmitter-containing SVs segregate and where peptide-containing LVs are also present. POMC and AgRP neurons also have non-synaptic boutons where only LVs are stored [5]. It has been proposed that, even in the case of non-synaptic release, neuropeptides could still act at postsynaptic sites [62]. It is, therefore, possible that α-MSH and AgRP released by Arc neurons at non-synaptic contacts modulate fast synaptic responses to neurotransmitters by binding to the MC4R localized to postsynaptic and peri-postsynaptic sites. Multiple GPCRs, such as the β1-adrenergic receptor (β1AR), serotonin 2A receptor (5-HT2AR), and dopamine 1 receptor (D1R), signal in response to the neurotransmitters epinephrine, histamine, and serotonin, respectively, and associate with PSD95 [63]. PSD95 interactions with GPCR have also been reported to modulate the binding of GPCRs to β-arrestins, endocytosis, and signaling [63].

We have found that the recycling of MC4R in the presence and absence of agonists is essential for maintaining receptor function in neuronal Neuro2A cells [22,64] and that lipid stress by exposure to elevated saturated fat or cholesterol depletion disrupts normal recycling properties of the receptor [19,65,66]. Future research is necessary to determine whether, under disease conditions, the inability of MC4R to reach PSD95/NMDAR sites impairs the ability of the receptor to modulate glutamatergic signaling. In addition, while this work finds localization of MC4R to postsynaptic sites, the question as to how MC4R reaches this location remains to be discovered. The system presented here includes primary hypothalamic neurons expressing endogenous MC4R and HA-MC4R, which replicates the MC4R localization observed in PVN and is a new tool for studying the effects of trafficking factors, disease, and drugs on the postsynaptic localization and function of MC4R.

## Data Availability

No new data were created.

## References

[1] Baldini G., Phelan K.D. (2019). The melanocortin pathway and control of appetite-progress and therapeutic implications. J. Endocrinol..

[2] Zhou Y., Chawla M.K., Rios-Monterrosa J.L., Wang L., Zempare M.A., Hruby V.J., Barnes C.A., Cai M. (2021). Aged Brains Express Less Melanocortin Receptors, Which Correlates with Age-Related Decline of Cognitive Functions. Molecules.

[3] Shen Y., Tian M., Zheng Y., Gong F., Fu A.K., Ip N.Y. (2016). Stimulation of the Hippocampal POMC/MC4R Circuit Alleviates Synaptic Plasticity Impairment in an Alzheimer’s Disease Model. Cell Rep..

[4] Machado I., Gonzalez P., Schioth H.B., Lasaga M., Scimonelli T.N. (2010). alpha-Melanocyte-stimulating hormone (alpha-MSH) reverses impairment of memory reconsolidation induced by interleukin-1 beta (IL-1 beta) hippocampal infusions. Peptides.

[5] Atasoy D., Betley J.N., Li W.P., Su H.H., Sertel S.M., Scheffer L.K., Simpson J.H., Fetter R.D., Sternson S.M. (2014). A genetically specified connectomics approach applied to long-range feeding regulatory circuits. Nat. Neurosci..

[6] Chen M., Wang J., Dickerson K.E., Kelleher J., Xie T., Gupta D., Lai E.W., Pacak K., Gavrilova O., Weinstein L.S. (2009). Central nervous system imprinting of the G protein G(s)alpha and its role in metabolic regulation. Cell Metab..

[7] Li Y.Q., Shrestha Y., Pandey M., Chen M., Kablan A., Gavrilova O., Offermanns S., Weinstein L.S. (2016). G(q/11)alpha and G(s)alpha mediate distinct physiological responses to central melanocortins. J. Clin. Investig..

[8] Lotta L.A., Mokrosinski J., Mendes de Oliveira E., Li C., Sharp S.J., Luan J., Brouwers B., Ayinampudi V., Bowker N., Kerrison N. (2019). Human Gain-of-Function MC4R Variants Show Signaling Bias and Protect against Obesity. Cell.

[9] Atasoy D., Aponte Y., Su H.H., Sternson S.M. (2008). A FLEX switch targets Channelrhodopsin-2 to multiple cell types for imaging and long-range circuit mapping. J. Neurosci..

[10] Fenselau H., Campbell J.N., Verstegen A.M., Madara J.C., Xu J., Shah B.P., Resch J.M., Yang Z., Mandelblat-Cerf Y., Livneh Y. (2017). A rapidly acting glutamatergic ARC→PVH satiety circuit postsynaptically regulated by alpha-MSH. Nat. Neurosci..

[11] Sumi T., Harada K. (2020). Mechanism underlying hippocampal long-term potentiation and depression based on competition between endocytosis and exocytosis of AMPA receptors. Sci. Rep..

[12] Wang X., Cui X., Li Y., Li F., Li Y., Dai J., Hu H., Wang X., Sun J., Yang Y. (2022). MC4R Deficiency Causes Dysregulation of Postsynaptic Excitatory Synaptic Transmission as a Crucial Culprit for Obesity. Diabetes.

[13] Wang Y., Bernard A., Comblain F., Yue X., Paillart C., Zhang S., Reiter J.F., Vaisse C. (2021). Melanocortin 4 receptor signals at the neuronal primary cilium to control food intake and body weight. J. Clin. Investig..

[14] Siljee J.E., Wang Y., Bernard A.A., Ersoy B.A., Zhang S., Marley A., Von Zastrow M., Reiter J.F., Vaisse C. (2018). Subcellular localization of MC4R with ADCY3 at neuronal primary cilia underlies a common pathway for genetic predisposition to obesity. Nat. Genet..

[15] Schou K.B., Pedersen L.B., Christensen S.T. (2015). Ins and outs of GPCR signaling in primary cilia. EMBO Rep..

[16] Nyamugenda E., Griffin H., Russell S., Cooney K.A., Kowalczyk N.S., Islam I., Phelan K.D., Baldini G. (2020). Selective Survival of Sim1/MC4R Neurons in Diet-Induced Obesity. iScience.

[17] Scheefhals N., Westra M., MacGillavry H.D. (2023). mGluR5 is transiently confined in perisynaptic nanodomains to shape synaptic function. Nat. Commun..

[18] Hentges S.T., Nishiyama M., Overstreet L.S., Stenzel-Poore M., Williams J.T., Low M.J. (2004). GABA release from proopiomelanocortin neurons. J. Neurosci..

[19] Griffin H., Sullivan S.C., Barger S.W., Phelan K.D., Baldini G. (2022). Liraglutide Counteracts Endoplasmic Reticulum Stress in Palmitate-Treated Hypothalamic Neurons without Restoring Mitochondrial Homeostasis. Int. J. Mol. Sci..

[20] Hansen K.B., Yi F., Perszyk R.E., Furukawa H., Wollmuth L.P., Gibb A.J., Traynelis S.F. (2018). Structure, function, and allosteric modulation of NMDA receptors. J. Gen. Physiol..

[21] Lobas M.A., Tao R., Nagai J., Kronschlager M.T., Borden P.M., Marvin J.S., Looger L.L., Khakh B.S. (2019). A genetically encoded single-wavelength sensor for imaging cytosolic and cell surface ATP. Nat. Commun..

[22] Mohammad S., Baldini G., Granell S., Narducci P., Martelli A.M., Baldini G. (2007). Constitutive traffic of melanocortin-4 receptor in Neuro2A cells and immortalized hypothalamic neurons. J. Biol. Chem..

[23] Muntean B.S., Zucca S., MacMullen C.M., Dao M.T., Johnston C., Iwamoto H., Blakely R.D., Davis R.L., Martemyanov K.A. (2018). Interrogating the Spatiotemporal Landscape of Neuromodulatory GPCR Signaling by Real-Time Imaging of cAMP in Intact Neurons and Circuits. Cell Rep..

[24] Sheng M., Kim E. (2011). The postsynaptic organization of synapses. Cold Spring Harb. Perspect. Biol..

[25] Fenske P., Grauel M.K., Brockmann M.M., Dorrn A.L., Trimbuch T., Rosenmund C. (2019). Autaptic cultures of human induced neurons as a versatile platform for studying synaptic function and neuronal morphology. Sci. Rep..

[26] Sampathkumar C., Wu Y.J., Vadhvani M., Trimbuch T., Eickholt B., Rosenmund C. (2016). Loss of MeCP2 disrupts cell autonomous and autocrine BDNF signaling in mouse glutamatergic neurons. Elife.

[27] Bekkers J.M., Stevens C.F. (1991). Excitatory and inhibitory autaptic currents in isolated hippocampal neurons maintained in cell culture. Proc. Natl. Acad. Sci. USA.

[28] Won S., Incontro S., Nicoll R.A., Roche K.W. (2016). PSD-95 stabilizes NMDA receptors by inducing the degradation of STEP61. Proc. Natl. Acad. Sci. USA.

[29] Sornarajah L., Vasuta O.C., Zhang L., Sutton C., Li B., El-Husseini A., Raymond L.A. (2008). NMDA receptor desensitization regulated by direct binding to PDZ1-2 domains of PSD-95. J. Neurophysiol..

[30] Wu X., Hammer J.A. (2021). ZEISS Airyscan: Optimizing Usage for Fast, Gentle, Super-Resolution Imaging. Methods Mol. Biol..

[31] Scheefhals N., MacGillavry H.D. (2018). Functional organization of postsynaptic glutamate receptors. Mol. Cell Neurosci..

[32] Roche K.W., Standley S., McCallum J., Dune Ly C., Ehlers M.D., Wenthold R.J. (2001). Molecular determinants of NMDA receptor internalization. Nat. Neurosci..

[33] Valtschanoff J.G., Weinberg R.J. (2001). Laminar organization of the NMDA receptor complex within the postsynaptic density. J. Neurosci..

[34] Goncalves J., Bartol T.M., Camus C., Levet F., Menegolla A.P., Sejnowski T.J., Sibarita J.B., Vivaudou M., Choquet D., Hosy E. (2020). Nanoscale co-organization and coactivation of AMPAR, NMDAR, and mGluR at excitatory synapses. Proc. Natl. Acad. Sci. USA.

[35] Lim I.A., Merrill M.A., Chen Y.C., Hell J.W. (2003). Disruption of the NMDA receptor-PSD-95 interaction in hippocampal neurons with no obvious physiological short-term effect. Neuropharmacology.

[36] Valtschanoff J.G., Burette A., Davare M.A., Leonard A.S., Hell J.W., Weinberg R.J. (2000). SAP97 concentrates at the postsynaptic density in cerebral cortex. Eur. J. Neurosci..

[37] Leonard A.S., Davare M.A., Horne M.C., Garner C.C., Hell J.W. (1998). SAP97 is associated with the α-amino-3-hydroxy-5-methylisoxazole-4-propionic acid receptor GluR1 subunit. J. Biol. Chem..

[38] Scott D.B., Michailidis I., Mu Y., Logothetis D., Ehlers M.D. (2004). Endocytosis and degradative sorting of NMDA receptors by conserved membrane-proximal signals. J. Neurosci..

[39] Shah B.P., Vong L., Olson D.P., Koda S., Krashes M.J., Ye C., Yang Z., Fuller P.M., Elmquist J.K., Lowell B.B. (2014). MC4R-expressing glutamatergic neurons in the paraventricular hypothalamus regulate feeding and are synaptically connected to the parabrachial nucleus. Proc. Natl. Acad. Sci. USA.

[40] Tolson K.P., Gemelli T., Gautron L., Elmquist J.K., Zinn A.R., Kublaoui B.M. (2010). Postnatal Sim1 deficiency causes hyperphagic obesity and reduced Mc4r and oxytocin expression. J. Neurosci..

[41] Granell S., Molden B.M., Baldini G. (2013). Exposure of MC4R to agonist in the endoplasmic reticulum stabilizes an active conformation of the receptor that does not desensitize. Proc. Natl. Acad. Sci. USA.

[42] Shinyama H., Masuzaki H., Fang H., Flier J.S. (2003). Regulation of melanocortin-4 receptor signaling: Agonist-mediated desensitization and internalization. Endocrinology.

[43] Bodzeta A., Berger F., MacGillavry H.D. (2022). Subsynaptic mobility of presynaptic mGluR types is differentially regulated by intra- and extracellular interactions. Mol. Biol. Cell.

[44] Nieuwenhuis B., Haenzi B., Hilton S., Carnicer-Lombarte A., Hobo B., Verhaagen J., Fawcett J.W. (2021). Optimization of adeno-associated viral vector-mediated transduction of the corticospinal tract: Comparison of four promoters. Gene Ther..

[45] Kugler S., Kilic E., Bahr M. (2003). Human synapsin 1 gene promoter confers highly neuron-specific long-term transgene expression from an adenoviral vector in the adult rat brain depending on the transduced area. Gene Ther..

[46] Nyamugenda E., Trentzsch M., Russell S., Miles T., Boysen G., Phelan K.D., Baldini G. (2019). Injury to hypothalamic Sim1 neurons is a common feature of obesity by exposure to high-fat diet in male and female mice. J. Neurochem..

[47] Lujan R., Nusser Z., Roberts J.D., Shigemoto R., Somogyi P. (1996). Perisynaptic location of metabotropic glutamate receptors mGluR1 and mGluR5 on dendrites and dendritic spines in the rat hippocampus. Eur. J. Neurosci..

[48] Kharazia V.N., Weinberg R.J. (1997). Tangential synaptic distribution of NMDA and AMPA receptors in rat neocortex. Neurosci. Lett..

[49] Racca C., Stephenson F.A., Streit P., Roberts J.D., Somogyi P. (2000). NMDA receptor content of synapses in stratum radiatum of the hippocampal CA1 area. J. Neurosci..

[50] Baude A., Nusser Z., Roberts J.D., Mulvihill E., McIlhinney R.A., Somogyi P. (1993). The metabotropic glutamate receptor (mGluR1 alpha) is concentrated at perisynaptic membrane of neuronal subpopulations as detected by immunogold reaction. Neuron.

[51] Nusser Z., Mulvihill E., Streit P., Somogyi P. (1994). Subsynaptic segregation of metabotropic and ionotropic glutamate receptors as revealed by immunogold localization. Neuroscience.

[52] Mango D., Ledonne A. (2023). Updates on the Physiopathology of Group I Metabotropic Glutamate Receptors (mGluRI)-Dependent Long-Term Depression. Cells.

[53] Moller T.C., Wirth V.F., Roberts N.I., Bender J., Bach A., Jacky B.P.S., Stromgaard K., Deussing J.M., Schwartz T.W., Martinez K.L. (2013). PDZ Domain-Mediated Interactions of G Protein-Coupled Receptors with Postsynaptic Density Protein 95: Quantitative Characterization of Interactions. PLoS ONE.

[54] Dunn H.A., Chahal H.S., Caetano F.A., Holmes K.D., Yuan G.Y., Parikh R., Heit B., Ferguson S.S.G. (2016). PSD-95 regulates CRFR1 localization, trafficking and beta-arrestin2 recruitment. Cell. Signal..

[55] Balthasar N., Dalgaard L.T., Lee C.E., Yu J., Funahashi H., Williams T., Ferreira M., Tang V., McGovern R.A., Kenny C.D. (2005). Divergence of melanocortin pathways in the control of food intake and energy expenditure. Cell.

[56] Chen Y., Lin Y.C., Kuo T.W., Knight Z.A. (2015). Sensory detection of food rapidly modulates arcuate feeding circuits. Cell.

[57] Shen Y., Fu W.Y., Cheng E.Y., Fu A.K., Ip N.Y. (2013). Melanocortin-4 receptor regulates hippocampal synaptic plasticity through a protein kinase A-dependent mechanism. J. Neurosci..

[58] Bernhem K., Blom H., Brismar H. (2018). Quantification of endogenous and exogenous protein expressions of Na,K-ATPase with super-resolution PALM/STORM imaging. PLoS ONE.

[59] Catsburg L.A., Westra M., van Schaik A.M., MacGillavry H.D. (2022). Dynamics and nanoscale organization of the postsynaptic endocytic zone at excitatory synapses. Elife.

[60] Wang H.Y., MacDonald M.L., Borgmann-Winter K.E., Banerjee A., Sleiman P., Tom A., Khan A., Lee K.C., Roussos P., Siegel S.J. (2020). mGluR5 hypofunction is integral to glutamatergic dysregulation in schizophrenia. Mol. Psychiatry.

[61] Aloisi E., Le Corf K., Dupuis J., Zhang P., Ginger M., Labrousse V., Spatuzza M., Georg Haberl M., Costa L., Shigemoto R. (2017). Altered surface mGluR5 dynamics provoke synaptic NMDAR dysfunction and cognitive defects in Fmr1 knockout mice. Nat. Commun..

[62] van den Pol A.N. (2012). Neuropeptide transmission in brain circuits. Neuron.

[63] Dunn H.A., Ferguson S.S. (2015). PDZ Protein Regulation of G Protein-Coupled Receptor Trafficking and Signaling Pathways. Mol. Pharmacol..

[64] Molden B.M., Cooney K.A., West K., Van Der Ploeg L.H., Baldini G. (2015). Temporal cAMP Signaling Selectivity by Natural and Synthetic MC4R Agonists. Mol. Endocrinol..

[65] Cragle F.K., Baldini G. (2014). Mild lipid stress induces profound loss of MC4R protein abundance and function. Mol. Endocrinol..

[66] McDaniel F.K., Molden B.M., Mohammad S., Baldini G., McPike L., Narducci P., Granell S. (2012). Constitutive cholesterol-dependent endocytosis of melanocortin-4 receptor (MC4R) is essential to maintain receptor responsiveness to alpha-melanocyte-stimulating hormone (alpha-MSH). J. Biol. Chem..

